# A Review of Contact Electrification at Diversified Interfaces and Related Applications on Triboelectric Nanogenerator

**DOI:** 10.1007/s40820-023-01238-8

**Published:** 2023-11-06

**Authors:** Jun Hu, Mitsumasa Iwamoto, Xiangyu Chen

**Affiliations:** 1 CAS Center for Excellence in Nanoscience, Beijing Institute of Nanoenergy and Nanosystems, Chinese Academy of Sciences, Beijing, 100083 People’s Republic of China; 2 School of Nanoscience and Engineering, University of Chinese Academy of Sciences, Beijing, 100049 People’s Republic of China; 3 Department of Physical Electronics, Tokyo Institute of Technology, 2-12-1 S3-33 O-Okayama, Meguro-Ku, Tokyo, 152-8552 Japan

**Keywords:** Contact electrification, Interfaces, Triboelectric nanogenerators, Diversified applications

## Abstract

The triboelectric nanogenerator (TENG) can effectively collect energy based on contact electrification (CE) at diverse interfaces, including solid–solid, liquid–solid, liquid–liquid, gas–solid, and gas–liquid. This enables energy harvesting from sources such as water, wind, and sound. In this review, we provide an overview of the coexistence of electron and ion transfer in the CE process. We elucidate the diverse dominant mechanisms observed at different interfaces and emphasize the interconnectedness and complementary nature of interface studies. The review also offers a comprehensive summary of the factors influencing charge transfer and the advancements in interfacial modification techniques. Additionally, we highlight the wide range of applications stemming from the distinctive characteristics of charge transfer at various interfaces. Finally, this review elucidates the future opportunities and challenges that interface CE may encounter. We anticipate that this review can offer valuable insights for future research on interface CE and facilitate the continued development and industrialization of TENG.

## Highlights

- The distinctive characteristics, underlying mechanisms, diverse range of selected materials, and modification methods of contact electrification (CE) at various interfaces are summarized and comparatively analyzed, offering valuable guidance for future investigations of triboelectric nanogenerator (TENG) at different interfaces.
- This review gives a detailed insight into the unique applications of TENG relying on different interfacial electrification.
- The challenges and development prospects of TENGs based on CE are discussed.

## Graphical abstract

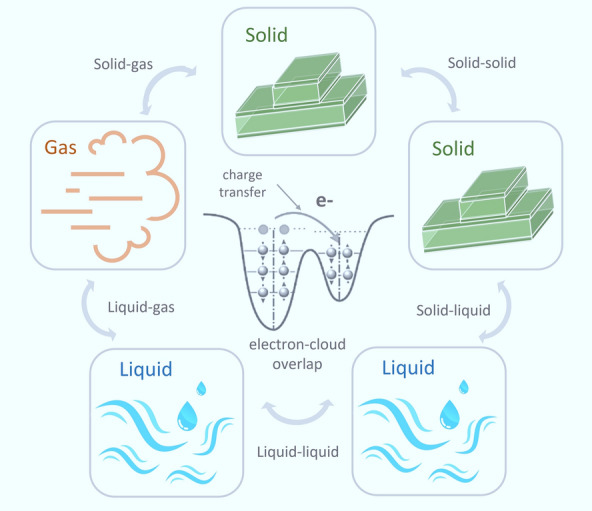

## Introduction

Contact electrification (CE) is a universal phenomenon that occurs on various interfaces [1]. This fact has been recognized by scientists since the discovery of static electricity in old days [2]. Over the years, many scientific and technological concepts regarding CE have been proposed. In 2012, the triboelectric nanogenerator (TENG) was introduced as a means to convert mechanical energy in the environment into electrical energy, by harnessing the CE effect to generate fundamental charge density [3]. This pioneering work has opened up new possibilities for achieving clean energy and self-powered sensory system. The TENG's operation can utilize the CE on a wide range of interfaces, including solid–solid [4, 5], liquid–solid [6–8], liquid–liquid [9], gas–solid [10], and gas–liquid interfaces [11]. Consequently, various TENG power units have been developed to collect energy [12] from water [13], wind [14], and even sound noise [15]. Additionally, self-powered sensor related to TENG can be applied to analyze diversified physical process occurring on various interfaces [16]. Meanwhile, the improvements of the energy conversion efficiency of TENG and the stability of the devices are closely dependent on the advancement of interfacial electrification studies [17]. Therefore, the development of TENG in various fields inspires continuous research interest in CE, and the progress in the study of CE at various interfaces can provide feedback for enhancing TENG performance, resulting in a virtuous research cycle [18].

With the development of TENG, a series of novel materials have been proposed for fabricating TENG [19], including ionized polymers [20], metallic glass [21], hydrogel [22] and various liquid electrification materials. These materials not only enrich the application scope of TENG but also bring out a series of unsolved interfacial issues for the study of CE [23]. Therefore, it is quite necessary to systematically summarize the mechanisms, regulation schemes, and unique applications of TENG based on different interfaces, to establish a complete physical understanding of TENG [24]. Previous reviews have focused on various aspects of TENG, including the CE mechanism at the interface [25], interfacial modification strategies [26], the triboelectric series [27] of different materials and so on. However, most of the reviews only focus on specific interface mechanisms or detailed functional device applications of TENG [28]. There is a lack of comparative analysis of TENG from the perspectives of diversified interfaces [29]. It is important to note that CE at different interfaces has distinctive mechanisms, and the related interfacial modification methods as well as the suitable application fields vary. Therefore, a comprehensive comparison of the different electrification characteristics at various interfaces is essential for the further development and industrialization of TENG [30].

This article takes a comparatively summary of the characteristics of different interface CE mechanisms as the entry point and provides a comprehensive review of the principles, ranges of material selection, methods of interface regulation, and applications of interface CE. Firstly, the article addresses the principles of CE at different interfaces, highlighting the superiority of the electron cloud model [5] and the hybrid EDL model [31], as well as the process of interfacial CE studies that inspires and complements each other. Secondly, the article offers an overview of the factors that affect CE at various interfaces and summarizes the general adaptation and targeted interface modification methods for different interfaces. Finally, the review discusses the application of each distinctive interface in various contexts. The article aims to offer a fresh perspective on the study of contact electrification at interfaces, emphasizing interface characteristics and interconnections between different interfaces. Its purpose is to stimulate innovative ideas and open new application domains that significantly enhance the efficiency of triboelectric nanogenerator (TENG) energy conversion. Additionally, the article discusses the opportunities and challenges for future studies on CE, aiming to provide insights for the development and innovation of related fields.

## Principle Mechanism for Contact Electrification at the Diversified Interfaces

### Principle of Contact Electrification at Solid–solid Surfaces

Solid–solid contact electrification is one of the most common modes of CE. To date, there have been numerous discussions relating to the nature of the charge generated in interfacial contact electrification [32, 33]. In recent years, various charge transfer models have been proposed to explain the process of solid–solid interface CE.

**Fig. 1:**
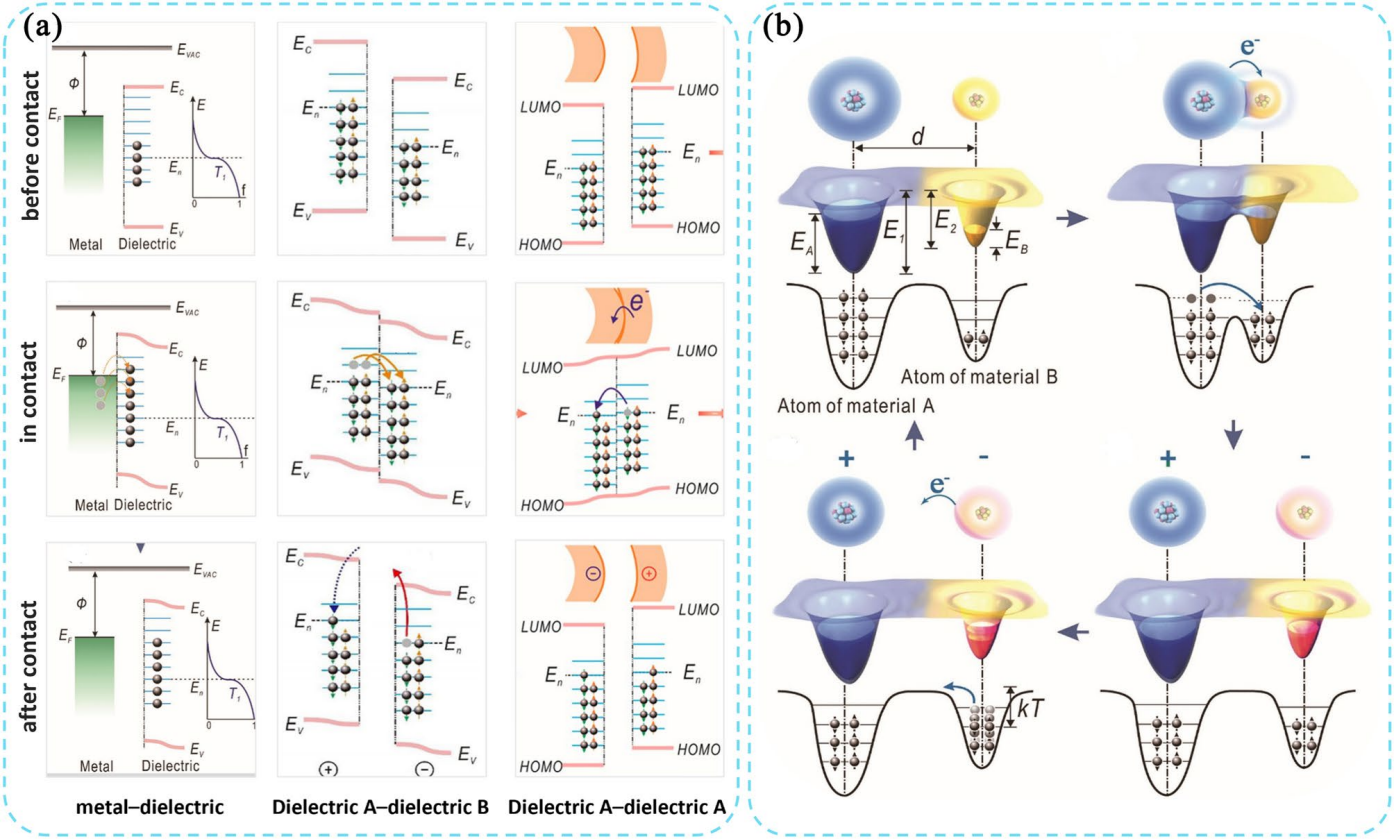
Contact electrification principle between solid and solid. **a** Principle of the charge transfer between different solid materials. **b** Electron-cloud–potential-well model for explaining release and transfer of charge between two materials

Figure 1a illustrates the energy bands between a metal and a dielectric [34], between two different dielectrics [5], and between the same dielectric in contact with each other [35]. Typically, the energy level occupied by electrons at the surface of dielectric material is lower than the Fermi level (EF) of the metal, and many surface states remain unoccupied. However, when the temperature reaches a certain point, some electrons in the metal gain enough energy to surpass the Fermi level. When the metal comes into contact with the dielectric material during this time, these high-energy electrons from the metal transfer to the unoccupied surface state of the dielectric material [34]. A similar situation may occur between different media [5]. This is due to the different ability of different dielectrics to retain charge. Although contact initiation usually occurs between two different materials, contact between two chemically identical materials can also generate electrostatic charges. In adjacent common dielectrics, the direction of charge transfer is influenced by the surface curvature of the sample. Positive curvature surfaces generally have a net negative charge, while negative curvature surfaces tend to have a net positive charge [35]. The stretching or compression of molecules on surfaces with different curvatures produces different surface energies, so that contact sites on surfaces with greater curvature can hold more electrons.

To account for the differences in energy band structure between metals and polymers, Wang et al. introduced an electron cloud/potential model. This model emphasizes charge transfer at the atomic level and visualizes the process of charge generation at the interface (Fig. 1b). In this model, atoms are depicted as potential wells with loosely bound electrons forming an electron cloud in specific orbitals [34]. Initially, electron transfer is hindered by the local capture effect of the potential well before contact between materials. However, when materials come into contact, overlapping electron clouds create an asymmetric double potential well due to differences in electronegativity. This asymmetry double potential well leads to variations in charge trapping capabilities and results in electron transfer. Due to the presence of the energy barrier, most of the transferred electrons will be retained after the materials have separated. Since potential barriers exist for all types of materials, this model is applicable to interfacial CE for all types of materials.

### Contact Electrification Principle Between Solid and Liquid

The electron cloud model provides a visualization of the electron transfer process at the atomic level [36]. This model is also applicable to explain the process of contact electrification at solid–liquid interfaces [37]. It demonstrates that electron transfer plays a dominant role in solid–liquid CE in some cases, in addition to the well-established interfacial ion transfer that has been known for decades [38].

**Fig. 2:**
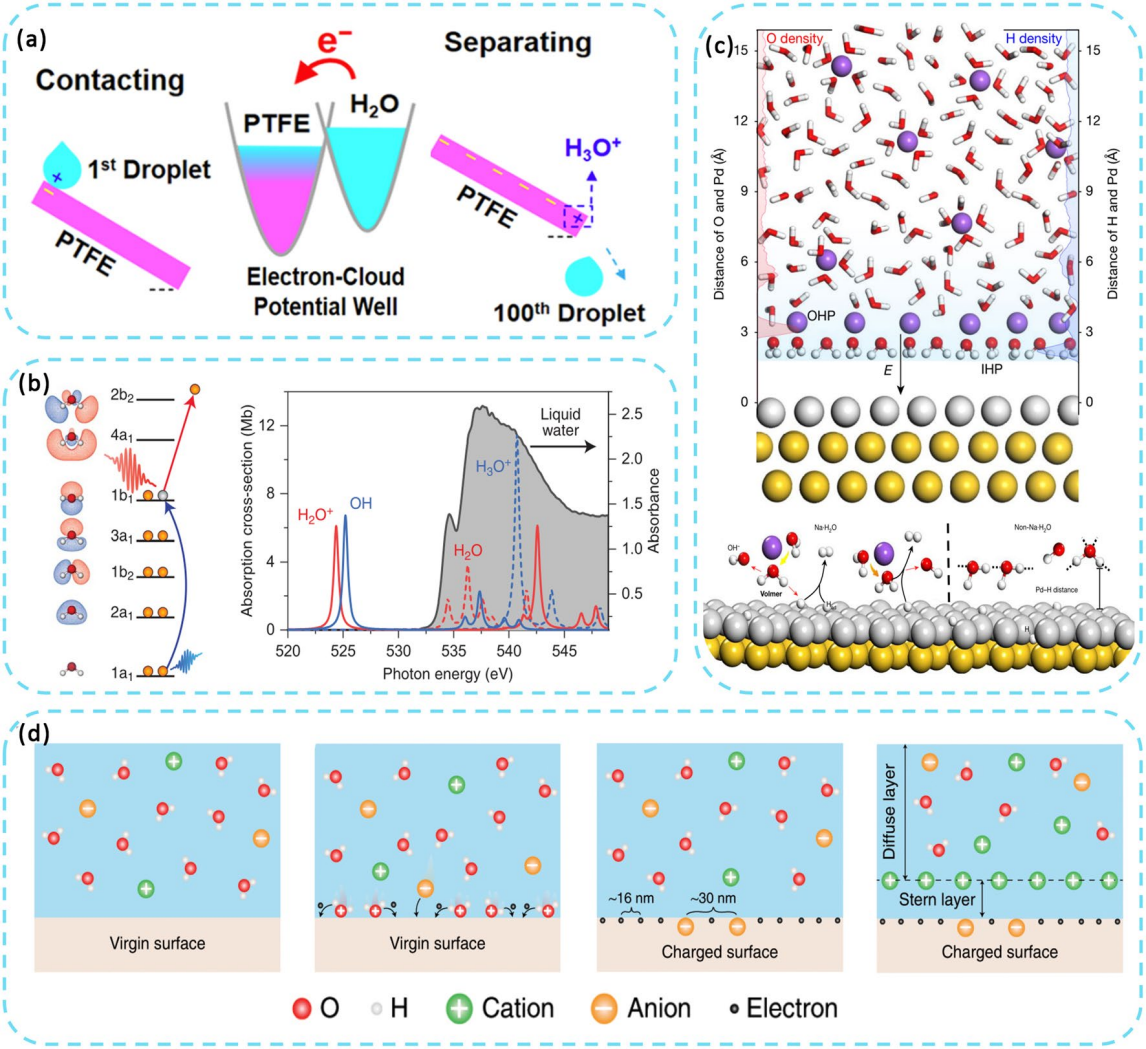
Contact electrification principle between solid and liquid. **a** Working mechanism and electron-cloud model of the liquid droplet TENG. **b** Primary chemical reaction following ionization of liquid water and proton transfer. **c** Schematic of the structure and dissociation of interfacial water. **d** Formation of electric double-layer at solid–liquid interface

A droplet-TENG was utilized as a probe to investigate interfacial charge transfer and examine dynamic saturation processes of charge accumulation and surface potential distribution on polymer surfaces, as shown in Fig. 2a [39]. In soaking–dropping experiments, the negative surface potential of the PTFE films remained relatively unchanged after immersion in different solutions, despite a four-order difference in the concentration of OH^−^ ion. The observation indicates a dominant role of electron transfer in solid–liquid contact electrification. To gain a deeper understanding of the generation of charge at the solid–liquid interface, attention was given to the main chemical reaction that occurs following the ionization of liquid water, i.e., H_2_O^+^  + H_2_O → OH + H_3_O^+^. As shown in Fig. 2b, the strong isolated soft X-ray absorption resonance (1a1 → 1b1) demonstrates the generation of electrons during the ionization of water molecules [40]. Additionally, studying interfacial water offers valuable insights in the structure and dynamic processes of water at the solid–liquid interface. As shown in Fig. 2c, surface interfacial water consists of hydrogen bonded and hydrated Na^+^ ionized water. The presence of structurally ordered interfacial water facilitates effective electron transfer across the interface [41].

In 2018, Wang et al. introduced a hybrid EDL model and a "two-step" process of formation, considering both electron transfer and ion adsorption, as shown in Fig. 2d. In the first step, due to the thermal motion and the pressure of the liquid, molecules and ions in the liquid impact the solid surface and electrons will be transferred between the solid atoms and water molecules due to the overlap of the electron clouds of the solid atoms and water molecules. The ionization reaction and ion adsorption occur simultaneously as parallel processes on the solid surface. It is worth noting that both the ions generated by the ionization reaction and the transferred electrons can change the potential distribution near the surface. In the second step, free ions in the liquid are attracted to the charged surface due to electrostatic forces. As a result, these ions migrate toward the charged surface, forming an EDL. In addition, the formation of EDL is influenced by the ability of the solid material to give/absorb electrons. This is consistent with the CE law between solids [31].

### Exploring Contact Electrification Principle at Other Interfaces

**Fig. 3:**
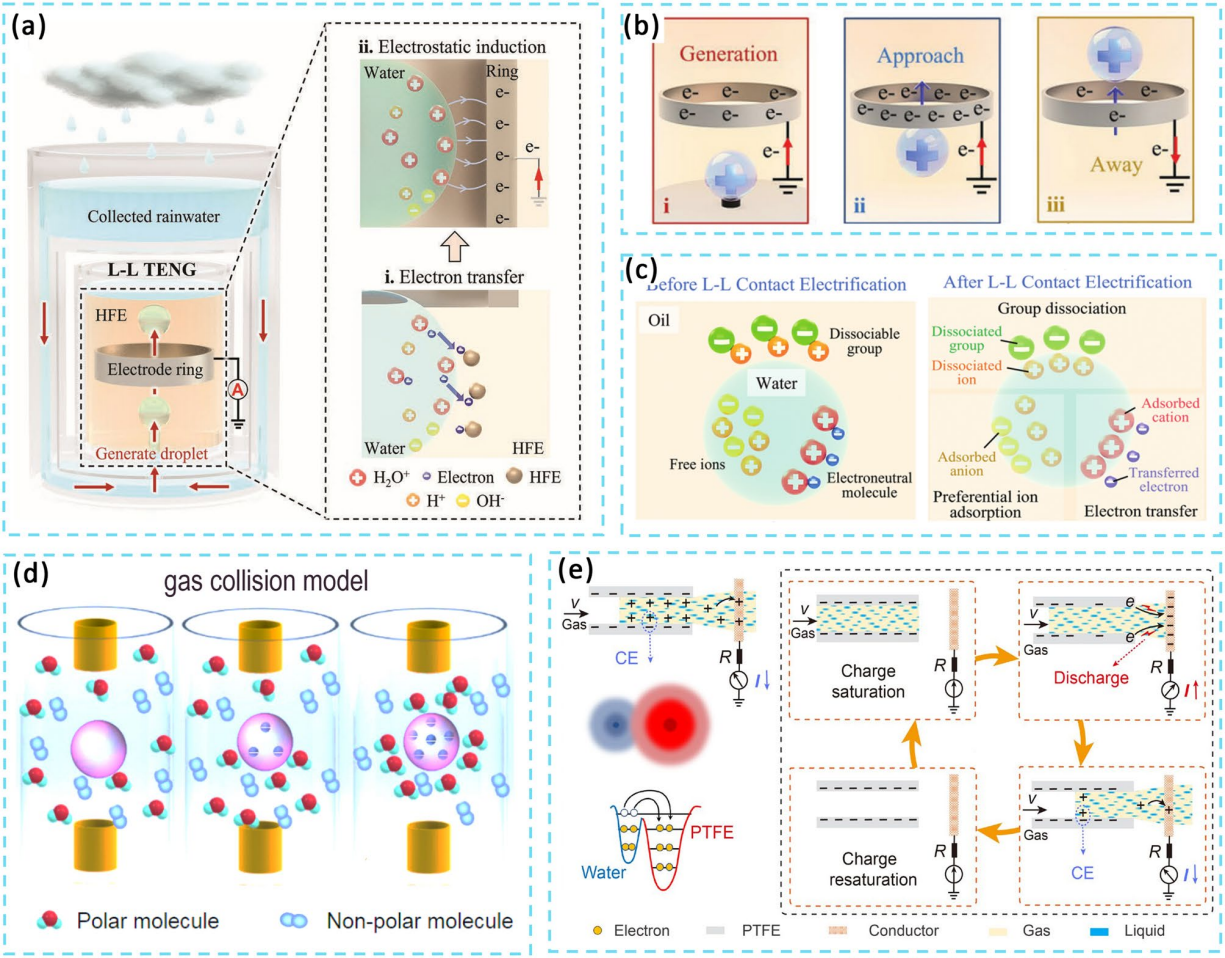
Contact electrification principle at other interfaces. **a** Illustration of the structure of liquid–liquid TENG and the electron transfer process. **b** The droplet movement process, including generating, approaching the electrode ring and moving away. **c** Mechanism of CE between liquid and liquid. **d** The gas collision model at the solid–gas interface. **e** Working principle of the gas–liquid TENG

**Fig. 4:**
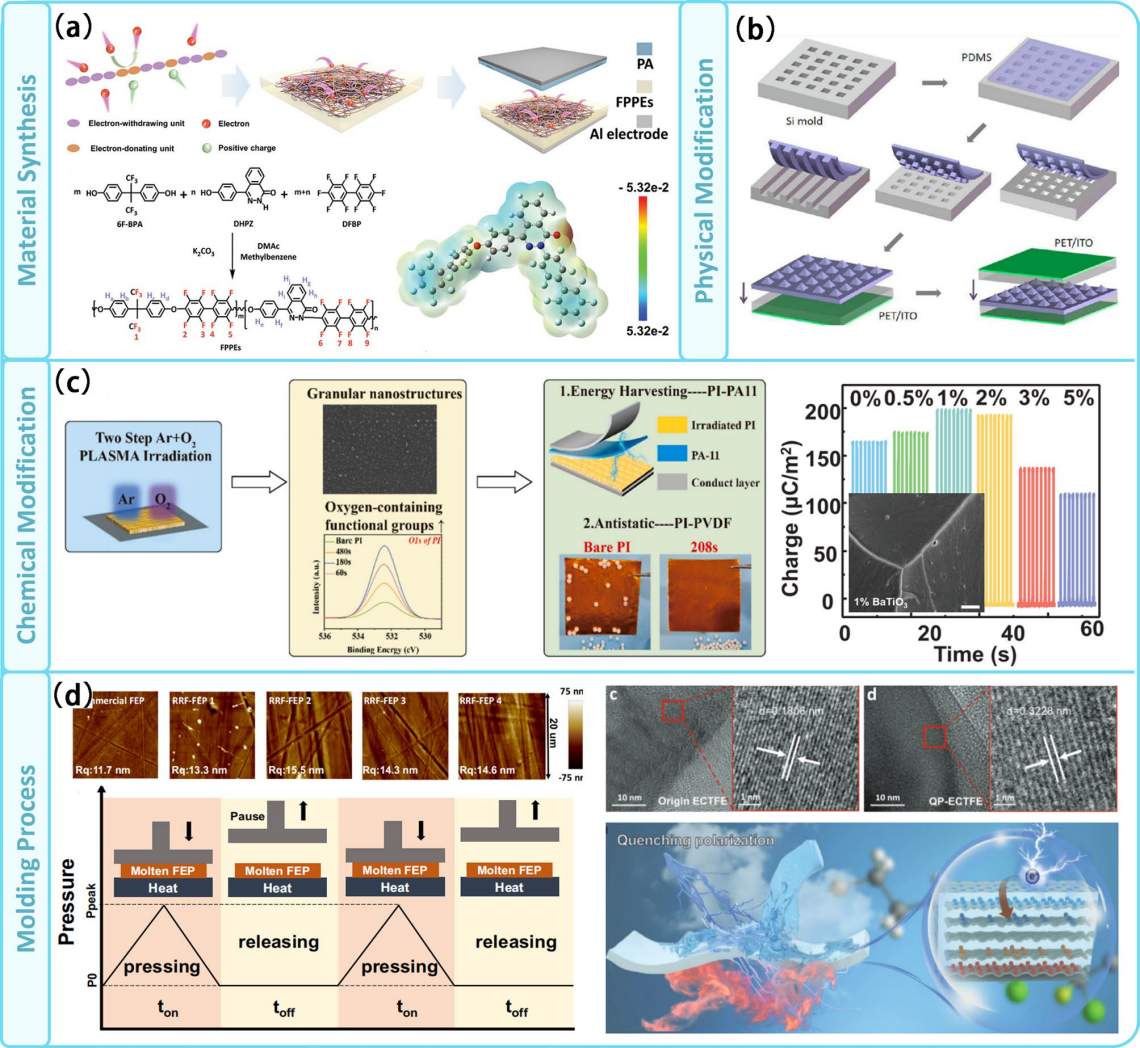
Modification for CE at solid–solid interface. **a** Molecular structure design and synthesis of materials. **b** Physical modification. **c** Chemical modification including ion irradiation and inorganic doping. **d** Special molding process to improve the CE properties of the material

In recent years, with the development of newer iterations in scientific detection techniques, there has been an increase in research into the contact initiation of electricity at other interfaces [42]. The study highlights the influence of H + on the dissociation of dominant groups and preferential adsorption of OH-, which plays a crucial role in influencing the coexistence mechanism of ζ-potentials in ion adsorption and electron transfer. By measuring the trends of the characteristic ζ-potentials of the Hex-water, OA-water and HFE-water systems at different concentrations of NaCl or HCl, it is possible to identify the dominant or co-existing mechanisms in the different systems. In the raw water system, the ζ-potential is initially negative due to the preferential adsorption of OH- ions at the interface. With the addition of HCl or NaCl, the OH- ions are gradually depleted by the neutralization of the H + ions, resulting in the aggregation of H + ions at the interface. As a result, the ζ-potential becomes positive. The magnitude of the negative ζ-potential initially increases and then decreases with increasing NaCl concentration, suggesting a competition between the salt effect and the compression of the bilayer. When HCl was added to the OA emulsion, the magnitude of the negative ζ-potential decreased as the introduction of H + ions hindered the dissociation of the carboxyl groups. These findings suggest that there is a coexistence mechanism in the OA-water system that involves preferential ion adsorption in addition to the dissociation of the main groups. In addition, this adsorption is affected by the concentration of H + in the system. Wang et al. presented a theoretical model of gas–solid CE, which incorporates a shooting gas collision model and illustrates the initial charge of the particles (Fig. 3d). Polar molecules were found to be attracted to the initial charge on the surface of solid particles. This attraction enhances gas–solid collisions. Given the limited presence of ions in gas–solid contact, it is reasonable to assume that gas–solid CE primary involves electron transfer. Also, the amount of transferred charge increases with surface area, distance travelled and the initial charge of the particle [10]. Additionally, the interaction between multiple state interfaces facilitates overcoming the limitations of the solid–solid and solid–liquid CE process. As shown in Fig. 4e, Wang et al. fabricated a Gas–liquid two-phase flow-based triboelectric nanogenerator. After water and PTFE come into contact, the gas–liquid two-phase flow rapidly replaces the air, filling the gap between the dielectric layer and the electrode. And the accumulated charge of the PTFE creates an electric field that penetrates the gas–liquid two-phase flow and generates a huge discharge. This effectively improves the problems of slow contact separation and small contact area in the solid–liquid CE process [11].

## Influence of Different Interface Conditions on the CE Process and Related Modulation Methods

Although CE has found a variety of ways for practical applications, further improvements in output performance are required to keep up with rapid development pace [43]. In this section, we focus on interfaces as a starting point and provide a comprehensive summary of the impact of various interface conditions on charge generation during CE. Furthermore, we explore different modulation techniques for enhancing CE at diverse interfaces.

### Material Selection and Modification Approaches for Contact Electrification at Solid–Solid Interfaces

In the solid–solid CE, the fatigue resistance and cyclability of the device are important indicators of the performance of the solid CE device in practical applications [44]. Wang et al. achieved long term operation with a high output of over 30,000 cycles by utilizing water-based graphene oxide as a lubricant [45]. However, the difference in electronegativity stemming from the inherent material molecular structure is the fundamental factor affecting the start of the charge [46]. Therefore, structural design and modification of materials are key factors in improving interfacial CE performance [47]. Common methods of regulation include designing of the material's molecular structure [48], implementing physical modifications [49], employing chemical functionalization [50], and enhancing process techniques.

Functional groups play a key role in determining the chemical properties of organic compounds. Moreover, they also have significant impact on the electronegativity of electrically frictional materials [51]. The selection of monomers containing suitable functional groups [52] and the control of monomer ratios [53] are the effective means of controlling the electronegativity of polymeric electrically charged materials. As shown in Fig. 4a, Tao et al. synthesized fluorinated poly(phthalazinone ether)s (FPPEs) by introducing phthalazinone moieties with strong electron-donating properties [54]. The materials containing 25% phthalazinone moieties exhibit a special crystalline behavior that allows them to trap and store more electrons. Fan et al. employed a physical etching process to prepare three regular polymer pattern arrays in the form of lines, cubes and pyramids. Notably, the devices with the pyramidal structure achieved an output voltage of up to 18 V, generating significantly more power compared to the unstructured films (Fig. 4b) [55]. Chemical modifications can cause direct changes in chemical bonding and are more commonly used in material modifications (Fig. 4c). Wang et al. used the surface treatment technique of plasma irradiation to increase the C–O and C–O content on the PI surface [56]. This was achieved by utilizing in situ two-step Ar + O_2_ reactive ion etching (RIE), which resulted in the formation of granular nanostructures on the PI surface. By doping the F-PI films with BaTiO_3_ nanoparticles, Wang et al. achieved the creation of electron deep traps and interfacial polarizations at interface, resulting in charge densities up to 200 µC m^−2^ and thermal charge stability up to 200 °C [57].

In addition, a well-designed processing process can also enhance the charge density for contact initiation. As shown in Fig. 4d, Liu et al. used repeated rheological forging to effectively modulate the surface functional group composition, crystallinity and dielectric constant of fluorinated ethylene propylene. By utilizing a forged 30-μm-thick film, an air breakdown mode TENG was capable of achieving an ultra-high charge density of 510 μC m^−2^ [58]. The team has also developed a quenched polarization (QP) method to generate ultra-high and long-lasting frictional charges on frictional electro-polymers with weak dipolarity. The QP films can achieve a charge density of 391 μC m^−2^, which is 200% higher than that of corona polarization. This method enables the use of charge storage materials that are not limited to highly insulating polymers [59].

Further research summarizes the factors at the microscopic level that influence the frictional starting properties of the polymer itself [58]. At the atomic level, the electronegativity of the atoms determines the electron-capturing capacity of the functional groups. This, in turn, influences the polarity and density of charges induced through friction [60, 61]. At the chain level, the molecular chain orientation plays a significant role [62]. It determines the probability of functional groups exposed in the surface region, coming into contact with the counter material, as well as the probability of the electron clouds overlapping [63]. Furthermore, higher crystallinity leads to an ordered arrangement of molecules, resulting in larger dipole moments and higher dipole polarization. The deep traps formed at the interface between the crystalline and amorphous regions enhance the storage of charge. In addition, material characteristics such as hardness [64] and surface roughness [65] and temperature difference [66] play a significant role in charge generation. It has been shown that from 0 to 145 K the output voltage, current, surface charge density, and output power increase by 2.7, 2.2, 3.0, and 2.9 times with the temperature difference between the hot and cold friction layers, respectively. And decrease as the temperature difference continues to increase. And also an increase in the hardness of the material usually reduces the contact radius, thus indirectly affecting the charge output.

### Factors Influencing the Solid–Liquid Interface and Related Modification Options

**Fig. 5:**
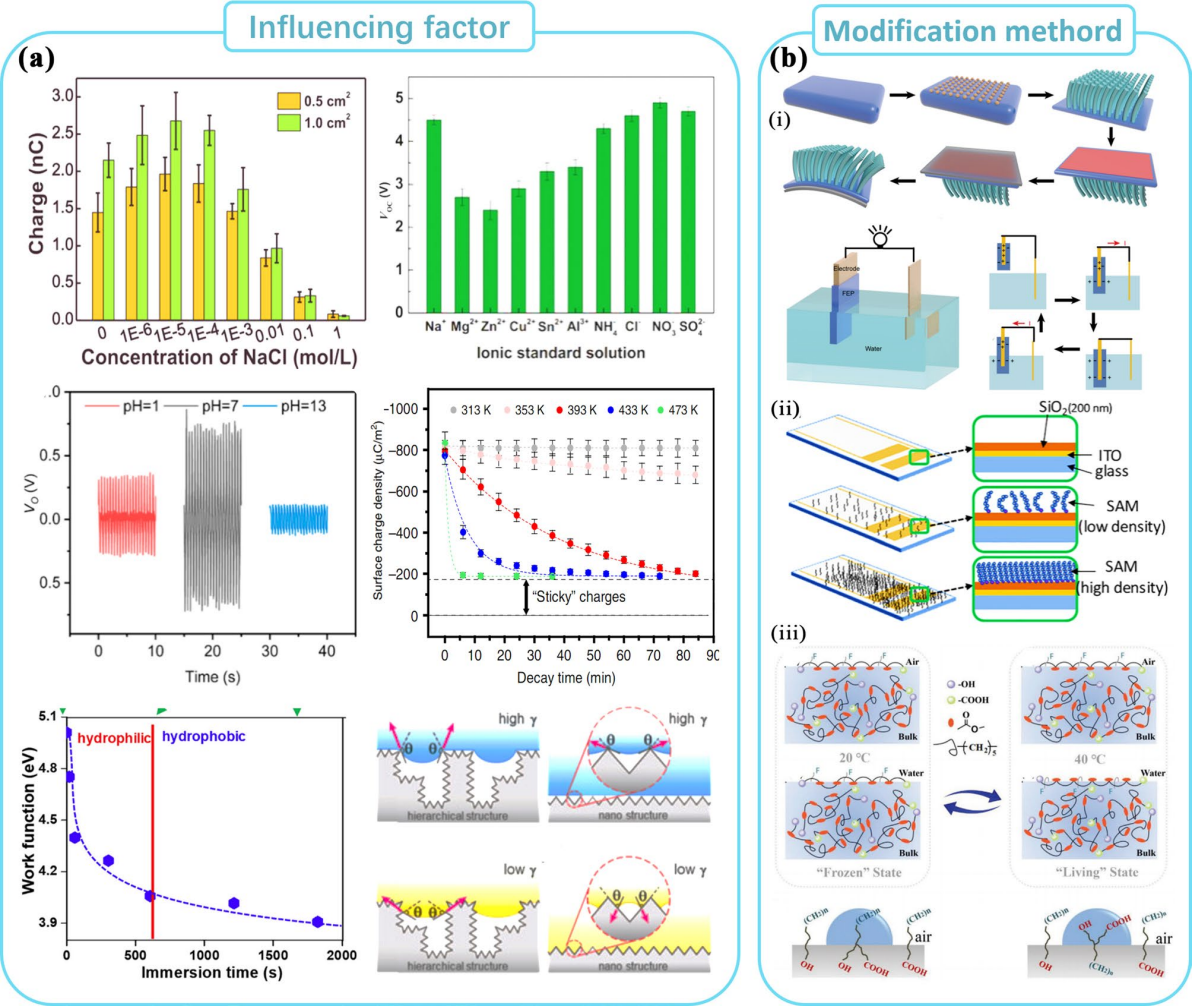
Factors and modification methods for CE between solid and liquid. **a** Factors affecting solid–liquid CE including ion concentration, ionic type, PH, temperature and surface tension and contact angle. **b** Modification for improving CE at solid–solid interface

Compared to solid–solid contact, ion adsorption plays a more significant role in solid–liquid CE [67]. As shown in Fig. 5a, different ion species [68], ion concentrations [69], and pH [70] of the liquid result in different contact voltages and effective contact charges. And similar to the solid–solid CE, the temperature difference [71] between the liquid and the solid also affects the occurrence of CE. In addition, due to the inherent liquid mobility, differences in contact angles due to the surface morphology of the solid and the hydrophilic and hydrophobic properties induced by different surface work functions play an important role in solid–liquid CE [72, 73].

Song et al. modified the surface from hydrophilic to hydrophobic by changing the density of the SAMs monolayer on the SiO_2_ surface, as shown in Fig. 5b. By partially exposing -OH groups on SiO_2_, they induced a high enough surface charge density to create a thicker EDL layer. The optimum contact angle and surface energy of the substrate were 95.5°and 30.7 mJ m^−2^, respectively [74]. Wang et al. achieved reversible PCL surface reorganization by controlling the temperature. The mobility of the PCL chains increased with temperature, causing the hydrophobic groups at the water-polymer interface to be switched by hydrophilic groups, resulting in a reversible electrical signal output [75]. Kim et al. has developed a comprehensive framework for liquid friction, encompassing electrolytes, organic solvents, oxidants, higher alcohols, and sugar alcohols. This framework outlines the chemical groups that commonly enhance or inhibit the liquid friction effect. For instance, hydroxyl groups are found to enhance the liquid friction effect, while the presence of phenyl groups inhibits the liquid tribological effect. This highlights the significance of functional groups in the solid–liquid charge extraction (CE) process [71].

### Emerging Approaches for Modification of CE at Other Interfaces

Regarding modification techniques for other contact interfaces, especially those involving gas–solid and gas–liquid interactions, there is still a lack of systematic research. This is primarily due to the constraints imposed by the limited availability of data and methodologies in these areas. Nevertheless, drawing on the insights gained from studies on solid–solid and solid–liquid interfaces, it is reasonable to conclude that altering the functional groups and molecular chain structure of the contact interface will remain a crucial approach for manipulating the electrical characteristics of other interfaces [76].

### Key Factors to Consider When Designing Different Interface Materials

**Fig. 6:**
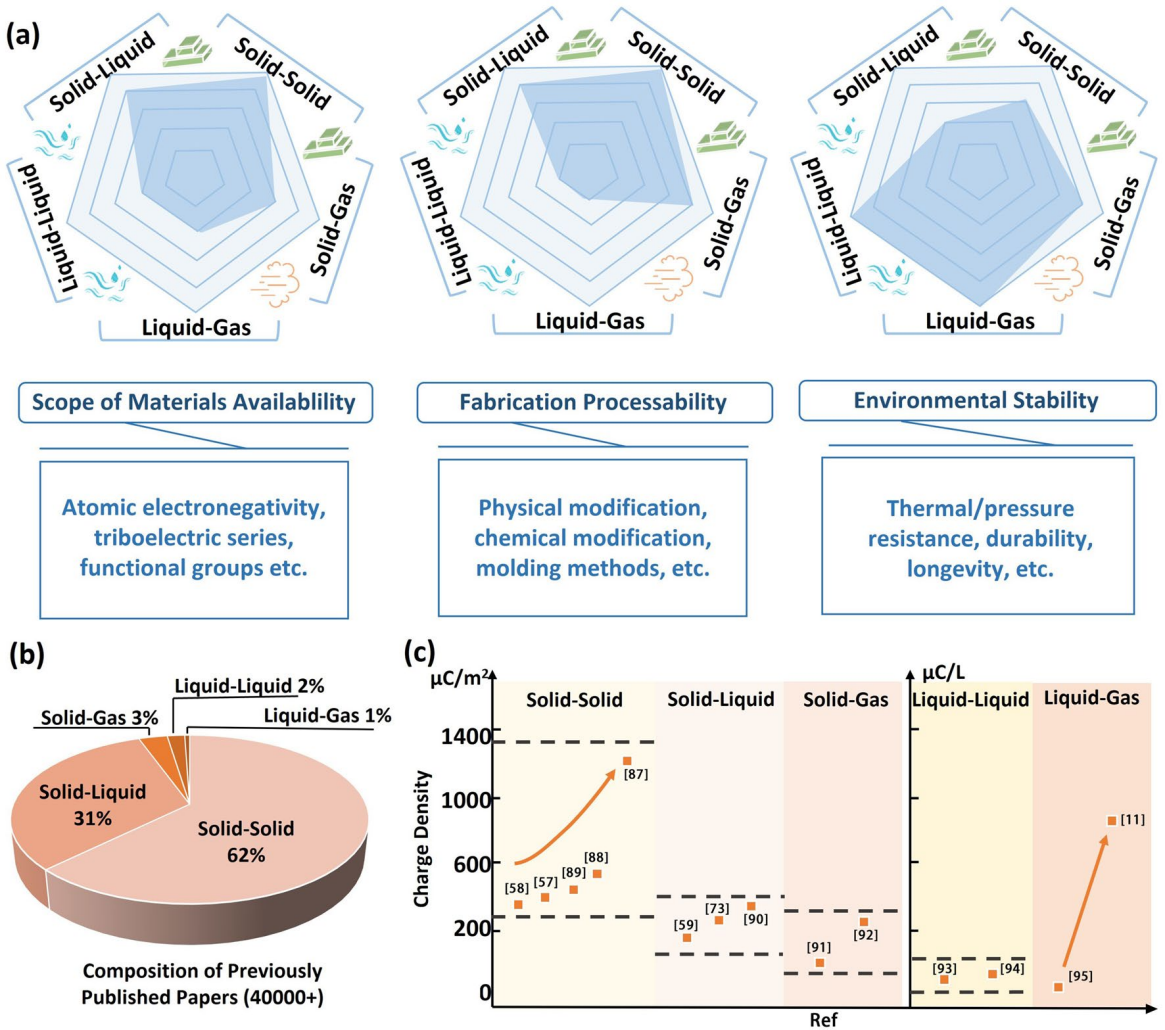
**a** Comparison of the range of materials available, processability and environmental stability for different interfaces. **b** Discussion of the exploitable prospects of CE at different interfaces based on number of existing studies and **c** limits of surface charge density

In the field of material selection for CE, the choice of materials is influenced by factors such as the electronegativity of atoms, the electronic series, and the type of functional group [77]. Solid materials, characterized by densely arranged atoms, provide ample opportunities for modifying chemical bonding and crystal structures. As a result, a wide range of material options with solid interfaces is available [78]. Additionally, the plasticity of solid materials grants them excellent processability, surpassing that of liquids and gases, as shown in Fig. 6a [79]. In addition, research on modifications of liquids has primarily focused on ionic species and concentrations, studies on gas modifications remain relatively scarce [80]. However, it is worth noting that unlike liquids and gases, solid materials are more susceptible to wear issues during friction and are prone to corrosion [81, 82]. Conversely, liquid–liquid and gas–liquid interfaces offer distinct advantages in terms of longevity and wear resistance.

Charge density is a crucial metric for evaluating energy conversion and is a dominant factor in trends observing across different interfaces [83]. However, it is important to note there is currently a significant variation in research progress for different interfaces [84]. As illustrated in Fig. 6b, this article examines the existing studies on CE at different interfaces, encompassing over 40,000 articles included in the statistical analysis. Of these, 62% of the studies focused on solid–solid CE, while 31% were for solid–liquid CE, indicating that previous research has primarily concentrated on these two types of interface CE. Only 3%, 2%, and 1% of the studies were for solid–gas, liquid–liquid, and liquid–gas interfaces, respectively. This may be due to technical limitations, but also suggests promising future developments [85]. The differences in the number of studies have resulted in variations in the methods used to increase charge density [86]. As shown in Fig. 6c, solid–solid CE produces the widest range of charge densities, with intrinsic charge densities of up to 1200 µC m^−2^ or more achievable thanks to the development of modification methods [57, 58, 60, 87, 88]. However, the various modification methods proposed for solids in previous studies have not increased the surface charge density of solid–liquid and solid–gas interfaces as much as desired, and the surface charge densities at these interfaces are mainly concentrated around 200 μC m^−2^ [59, 73, 89–91], which may be due to the weak charge-binding ability of liquids and gases. The same is true for liquid–liquid CE [92, 93]. However, it is noteworthy that high surface charge densities have been achieved for liquid–gas interface CE, where the gas–liquid two-phase flow approach has successfully increased the charge density range from a few tens to 800 µC L^−1^ [11, 94]. This exceptional phenomenon at special interfaces highlights the impressive progress of special interface CE.

## Applications Related to Contact Electrification at Different Interfaces

Frictional electrical devices that are controlled by functionalized interface structures of different natures have found widespread use in the fields of physics, chemistry and biology [95, 96]. Moreover, energy conversion and transmission based on the frictional electric effect have been extensively employed in wearable devices [97], biomedical applications [98], smart homes [99], intelligent transportation [100, 101], environmental monitoring, and other domains [102]. These application scenarios not only enhance the energy efficiency of devices [103] but also provide greater convenience and comfort in people's lives. This session describes the practical application of different contact interfaces for starting electrical incoming bands in terms of their characteristics.

### Diversified Application Design Based on Solid–solid Contact Electrification

**Fig. 7:**
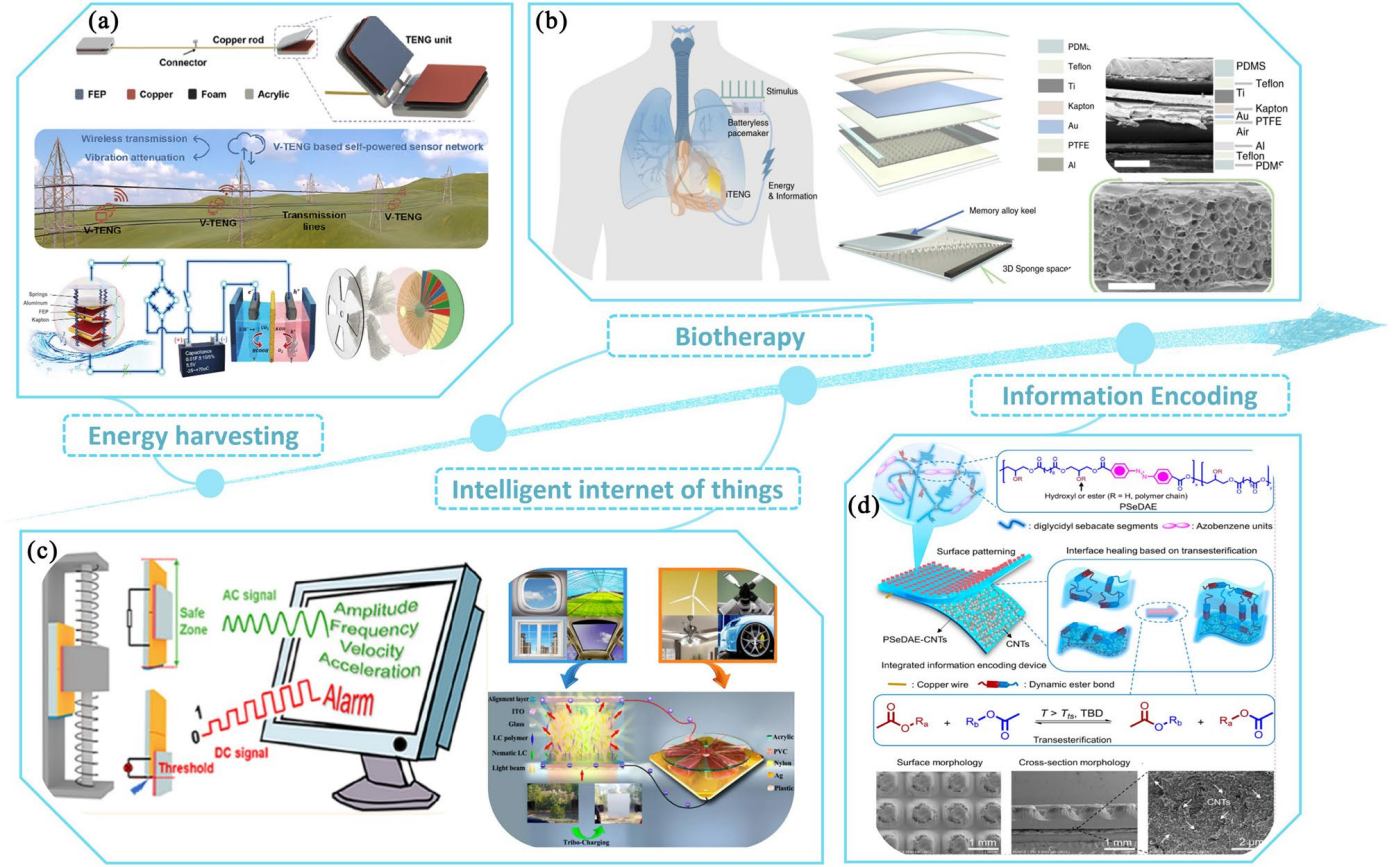
Application scenarios derived from the diverse solid–solid interface CE including **a** energy harvesting, **b** biotherapy, **c** intelligent internet of things and **d** information encoding

Solid materials are generally rigid and resistant to external influences that can change their shape or volume [104]. As a result, solid interfaces can be diversified to suit different application scenarios and needs [105]. Currently, a variety of interface CE forms such as single-layer [106, 107], multi-layer [108–110], bending [111] and turntable [112] have been developed and are used in a variety of environments. Energy harvesting in many conditions is one of the key applications of TENG [113]. Wu et al. designed and fabricated a hybrid energy vibration-driven triboelectric nanogenerator, which uses wind-driven TENG to generate contact separation and thus harvest vibration energy for use as a power source in self-powered information detection/transmission/alarm systems [114]. Jr-Hau He presented a wave energy-driven electrochemical system to maximize the production of formic acid using the energy collected from the nanogenerator, producing 2.798 mmol of formic acid per day from wave energy collected over an area of 0.04 m^2^, with a CO conversion efficiency close to 100% [115], as shown in Fig. 7a. Tao Jiang has developed a highly stable multi-phase TENG that achieves a high average output power DC output under constant current conditions by means of electrode misalignment and circuit connection using common everyday materials, expanding the choice of TENG materials and using its rotational characteristics for cycling runner energy harvesting [116]. Figure 7b describes a fully implantable symbiotic pacemaker (SPM) based on an implantable frictional electrical nanogenerator, where the pacemaker and the body form an interconnected symbiotic system where the SPM takes in biomechanical energy from cardiac motion, respiratory motion and blood flow while the body receives electrical stimulation from the SPM to regulate cardiac physiological activity. The device has been successfully implemented in large animal models for cardiac pacing and sinus arrhythmia correction, and has the advantages of excellent output performance, high power density and durability [117].

In the era of 5G and the Internet of Things (IoT), integrated systems for TENGs and IoT technologies are developing rapidly and are dramatically changing the way humans produce and live their daily lives [118]. As shown in Fig. 7c, Wang et al. introduced a self-powered vibration sensor system using dual-mode TENG. Once the vibration amplitude exceeds the danger threshold, AC immediately converts to DC and simultaneously triggers the alarm system directly. This device can detect the vibration characteristics of a building structure in real time, accurately predict construction hazards and monitor the structural health of the building [119]. Wang et al. developed a self-powered normally transparent smart window by combining a rotating freestanding sliding frictional electro-nanogenerator (RFS-TENG) and a polymer network liquid crystal (PNLC) cell to achieve ultra-transparent and ultra-hazy state switching with a response time of less than 7 ms. The window can be used in applications such as self-powered skylights and wind-driven intelligent agricultural systems [120]. Information encoding devices are an important branch of the Internet of Things, in areas such as smart defense systems and improvised explosive devices (Fig. 7d) [121]. You et al. investigated an elastomer with triple-shape-memory effect and integrated the development of a multifunctional self-powered information encoding device (IED). The IED has independent, spatio-temporal reprogrammable, self-healing and erasable properties.

**Fig. 8:**
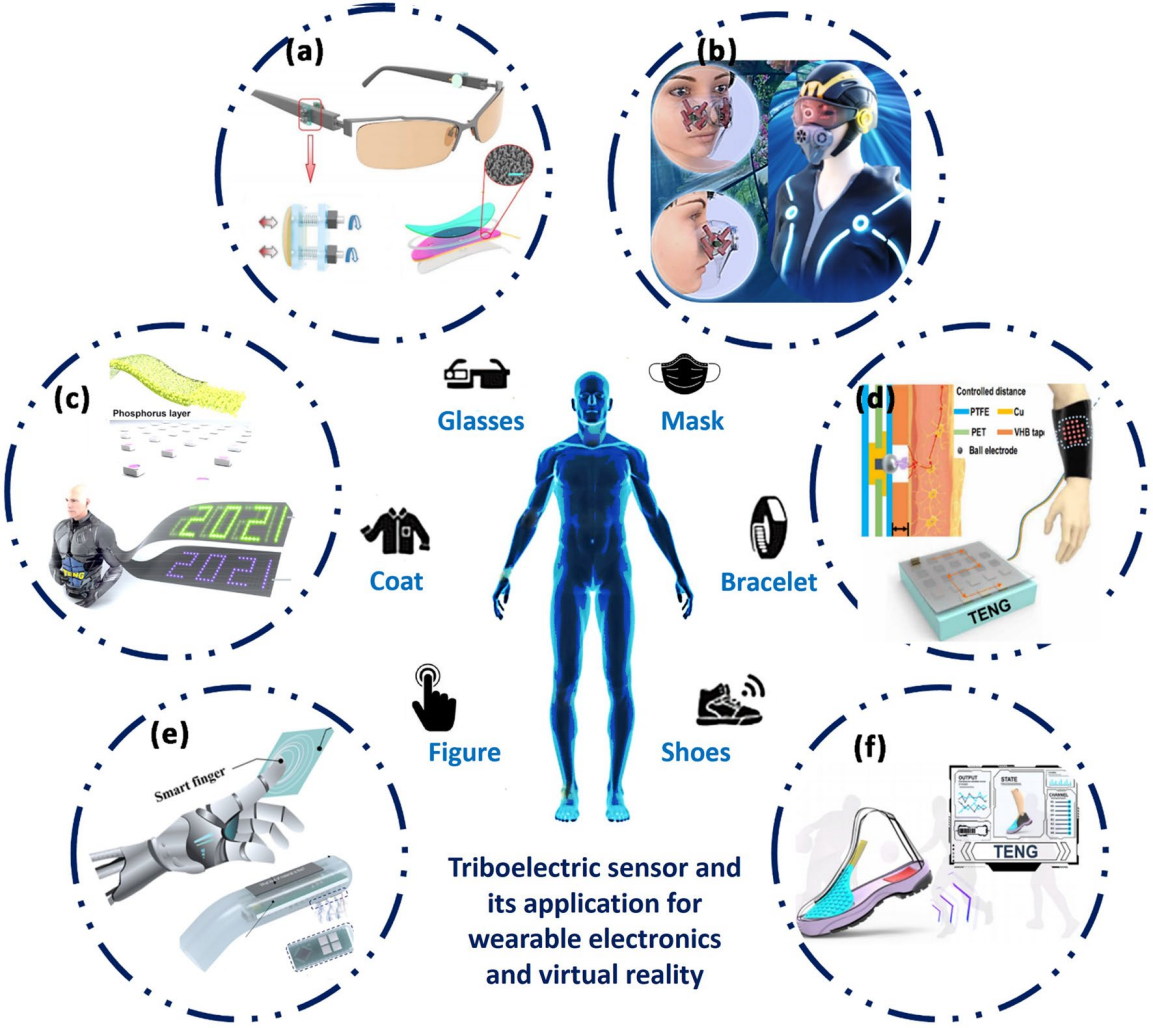
Triboelectric sensor provides a boost to wearable devices and virtual reality development. **a** Self-powered eye movement monitoring system. **b** Self-powered virtual olfactory generation system. **c** Nanogenerator with self-powered persistent phosphorescence for reliable optical display. **d** Self-powered electro-tactile system. **e** Artificial tactile perception smart finger. **f** Monitoring the degree of comfort of shoes using triboelectric pressure sensors

As an important product of internet development, virtual reality (VR) and augmented reality (AR) aim to enable the input and output of analogue signals for multiple senses such as sight, smell and touch, providing a near-realistic virtual experience for humans. The high voltage, low current and wide range of material options of wearable TENG devices are facilitating the manufacture of VR and AR devices [122, 123]. Pu et al. reports a TENG-based micromotion sensor that can effectively capture blink motion at super-high signal level (~ 750 mV) and can be applied to wireless hands-free typing systems (Fig. 8a) [124]. Yang et al. used electrostatic field-accelerated evaporation to achieve virtual olfaction generation, and designed their system to achieve a volatile liquid spray flow rate of 0.1 μL s^−1^ and an average evaporation rate of 0.12 mg s^−1^, allowing the user to perceive odor generation within 3 s (Fig. 8b) [125]. Shi et al. designed self-powered, painless, highly sensitive virtual haptic sensors that can be used for virtual tactile displays, Braille guidance, and neural stimulation [126]. The team combined TENG with ultra-long organic phosphorescence (UOP) and elastomers to enable visual tracking of electrical signals [127] (Fig. 8c, d). By further incorporating intelligent algorithms, it is possible to achieve systems that surpass human tactile perception. The artificial tactile smart finger designed by Xuecheng Qu et al. can accurately identify material type and roughness with an accuracy of 96.8% by integrating frictional electrical sensing and machine learning (Fig. 8e) [128]. In addition, similar systems can be used to monitor the comfort of shoes during exercise, informing sports training and the custom design of shoes (Fig. 8f) [129]. The sensor units embedded within the in-shoe sensor pad are airbag TENGs composed of activated carbon/polyurethane (AC/PU) and microsphere array electrodes. This design enables an exceptionally broad detection range, allowing the sensor to handle shock pressures across various scales. Furthermore, In addition, the magnetically coupled inductive wireless transmission system effectively extends the battery life of the sensor. This offers long-lasting information into abrupt variations in foot pressure and friction, which can often go unnoticed by the user.

### Applications Based on CE Between Solid and Liquid

**Fig. 9:**
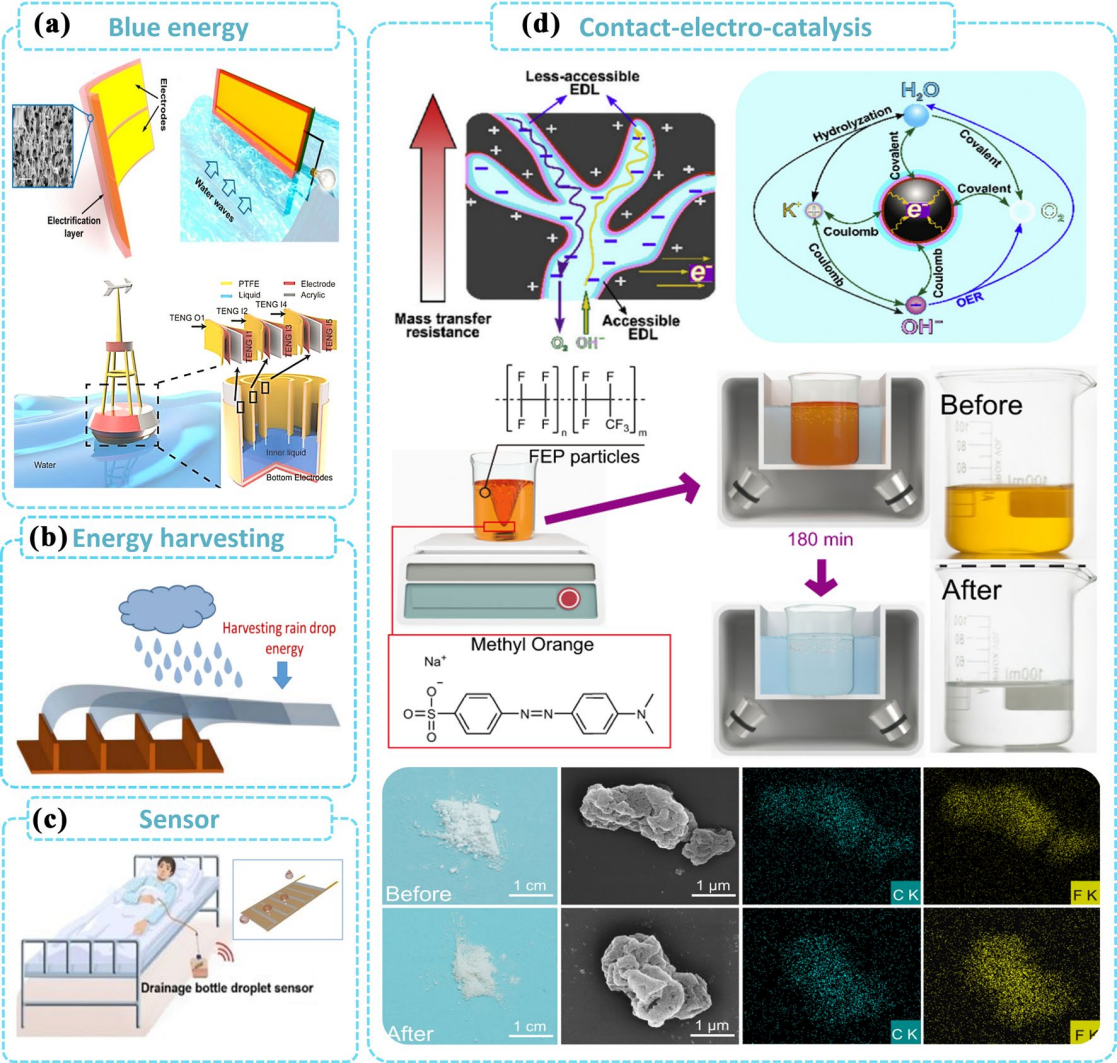
Applications of TENG using CE between solid and liquid including **a** blue energy, **b** raining energy harvesting, **c** self-powered sensor and **d** electrocatalysis

Blue energy refers to renewable energy sources derived from the ocean [130]. Compared to conventional energy sources, blue energy does not produce greenhouse gases and other pollutants and has a lower impact on the environment and ecosystems, while also being able to reduce reliance on limited fossil energy sources [131, 132]. Such environments which contain large amounts of water resources, such as oceans, rivers and rainwater [133], provide an ample liquid matrix for solid–liquid power generation and can take full advantage of the advantages of power generation at the solid–liquid interface. As shown in Fig. 9a, the study reports an integrated solid–liquid TENG based on a fluorinated ethylene propylene film. The device results in an electrical output efficiency of 7.7%, capable of harvesting energy from wave water during surfacing and sinking [134]. Pan et al. designed the buoy L-S TENG, which has 48.7 times more friction and energy output than a solid–solid contact frictional electro-electric nanogenerator of the same area, to harvest energy from low frequency motions such as up and down, vibration and rotation. the L-S TENG network can achieve an output of 290 µA, 16 725 nC and 300 V with a single trigger pulse to generating multiple continuous damping signals to maximize the energy requirements of SOS transmitters for marine emergencies [135]. Figure 9b shows a TENG using shape memory materials to harvest energy from raindrops with a power density of up to 230 mW m^−2^ under rainy conditions [136]. In addition to water energy harvesting, solid–liquid CE exists in a wide range of applications in liquid sensing [137]. The superhydrophobic liquid–solid TENG as a biomedical droplet sensor is repulsive to a wide range of solutions including blood, enabling real-time monitoring of clinical drainage operations and intravenous infusions (Fig. 9c). Solid–liquid CE is also often used in combination with chemical reactions such as electrochemistry [138], catalysis [139–141], colloidal suspension [142], prevention of adsorption and corrosion [143]. As illustrated in Fig. 9d, contact electrocatalysis (CEC) is able to use the electrons exchanged at the interface between water and dielectric powder for chemical reactions, acting on refractory organic compounds, resulting in a novel wastewater treatment system [139]. Experiments showed that 50 mL of 5-ppm aqueous solution of methyl orange (MO) was completely degraded after 3 h under the aid of 20 mg of pristine FEP powder and ultrasound. It also proved that contact electrocatalysis can effectively enrich the variety of catalytic mechanisms and broaden the range of material options for catalysts [144].

## Highlighting Unique Applications of Contact Electrification in Other Interfaces

**Fig. 10:**
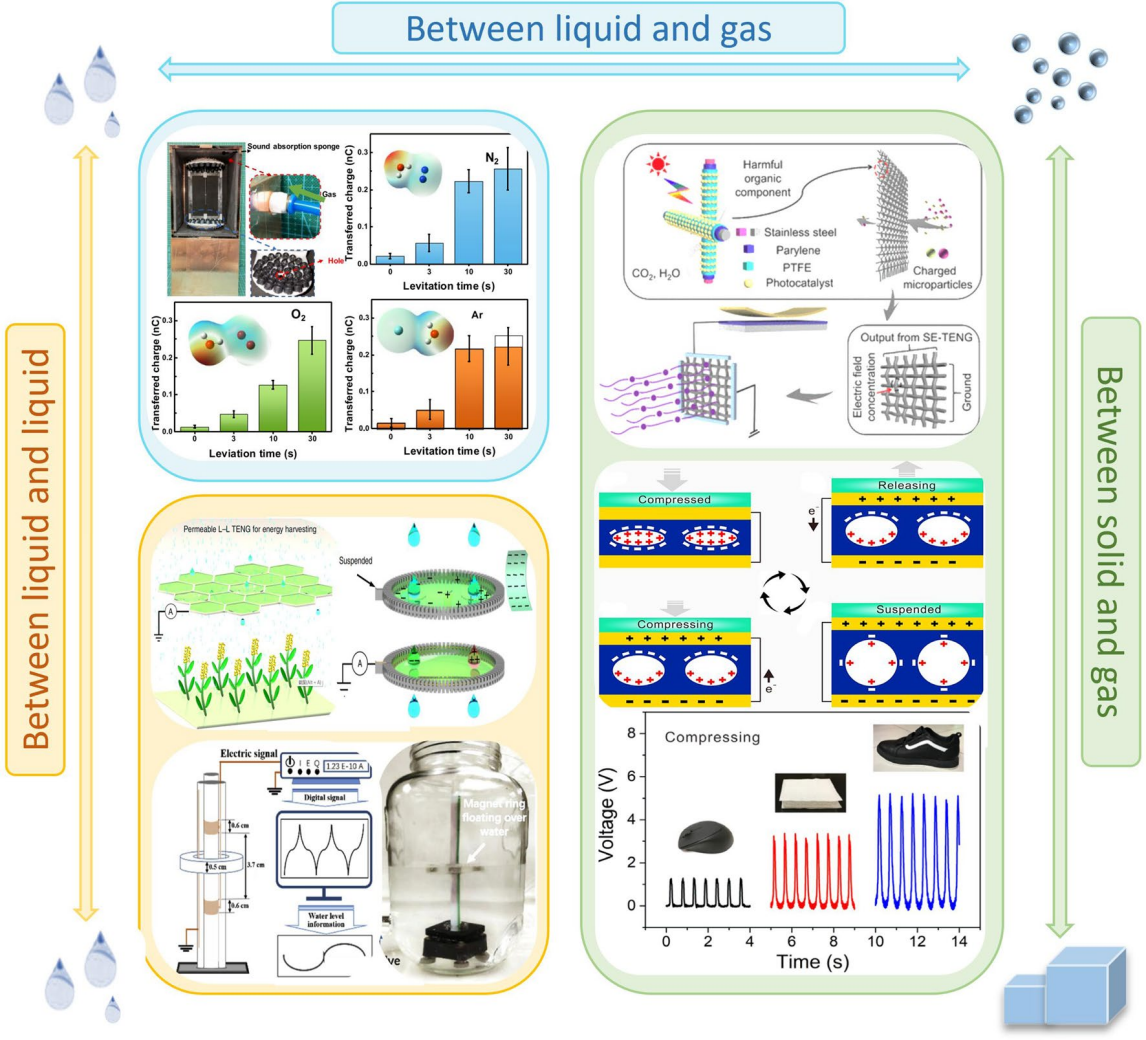
Applications of TENG based on contact electrification at other interface

The problem of easy merging and difficult separation at the liquid–liquid interface contact is still limited by the inadequacy of existing technology and there is a lack of practical applications [145]. Chen et al. established an L-L TENG for liquid energy harvesting using the interaction between liquid droplets and separate liquid films [92]. As shown in Fig. 10, a droplet with a volume of about 40μL generates a peak power of 137.4 nW, and the output power can be further increased by using a multi-membrane system. In addition, negligible friction at the L-L interface results in minimal energy loss compared to the solid–solid or liquid–solid interfaces, allowing for the collection of mechanical energy including in raindrops, irrigation currents, and microfluids without blocking or capturing moving objects [1]. Wang et al. introduced a lubricant layer between the ferromagnetic fluid and the substrate to form a liquid–liquid frictional-electric interface, thus pushing the limits of motion velocity measurement for TENG as a velocity sensor and demonstrating its potential application as a self-powered water/liquid level sensor [146]. Taking inspiration from the phenomenon of electrostatic charges and lightning generation in suspended droplets within clouds, Fan Wang et al. devised an innovative approach using acoustic levitation to suspend and rub liquid droplets against air. This process resulted in the acquisition of a positive charge on the droplet surface through contact electrification (CE) at the liquid–air interface [94]. Zhang et al. investigated the electrostatic process of microdroplets at the gas–liquid interface. A series of work on microdroplet catalytic reactions is carried out using a unique home-built field-induced droplet ionization mass spectrometry methodology. Experiments on spontaneous oxidation of dozens of organic molecules shows that the electrification at the gas–liquid interface is of great value in the field of microdroplet catalysis [147].

Due to the rapid development of industry and fuel vehicles in cities, atmospheric pollution caused by particulate matter or volatile organic compounds (VOCs) has become a serious threat to human health. The static electricity generated by the interface between solids and gases can be used to achieve gas treatment and purification. Li et al. combine TENG and photocatalytic technology to remove pollutants from indoor air. The TENG is able to generate a strong electric field of over 1100 V on the filter network, doubling the degradation efficiency of both RhB and formaldehyde in the same amount of time [90]. A porous elastomeric PDPU with ultra-high static surface potential and excellent compressibility was designed to generate electrical energy through periodic compression. This self-healing elastomer can be adhered to other substrates and is compatible with substrates including textiles, shoes, computer mouse devices and keyboards to harvest mechanical energy during the compressive motion [91].

### Expand the Scope of Application Based on CE at Different Interface

TENG controlled by functionalized interface structures of different natures are widely used in multiple areas. However, the degree of exploitation of different interfaces in different application scenarios varies due to their respective characteristics [148]. Table 1 illustrates the degree of application of different interface CE in common fields. TENG have great advantages as passive devices in the field of self-powered energy packs [149, 150], and all interface CEs are capable of energy supply in the field of wearable energy packs [98]. However, for the manufacture of implantable energy packs, solid shape stability and plasticity are necessary to ensure device safety and stability, making devices based on solid–solid CE the most widespread [96]. While solid–liquid CE can also be realized using blood and tissue fluids in the human body. Research in the biomedical field also focuses more on solid–solid and solid–liquid interfaces. In the field of blue energy, the abundance of water in the ocean makes solid–liquid CE dominant [151, 152]. Self-powered sensors are a universal application for CE at different interfaces. Self-powered sensors based on solid–solid interfaces can be used to monitor [153] a variety of human movements for signal transmission [154] and integrated with smart IoT devices [155], while those based on solid–liquid and solid–gas interfaces are more commonly used to monitor environmental conditions. In addition, TENG based on specialized interfaces offer an enhanced capability for precise sensing in specific conditions. Additionally, CE processes at the solid–solid and solid–liquid CE can be used for data processing [156]. It can be observed that solid–solid CE, which has been studied the longest and has the best underlying theory, also has the most application scenarios. However, other states of matter have their own strengths, such as the unique application of solid–liquid CE in catalysis [140], and interfaces containing gases have a unique advantage in air purification [157].

**Table 1:** Application degree of various interface CE in common fields

	Solid–solid	Solid–liquid	solid–gas	Liquid–Liquid	Liquid–gas
Wearable energy package	√	√	√	√	√
Implantable energy package	√	√			
Blue energy harvesting	√	√		√	
Diversified sensor	√	√	√	√	√
Data acquisition/transfer	√	√			
Biomedicine	√	√			
Electrocatalysis		√			
Air purification	√		√		√

**Fig. 11:**
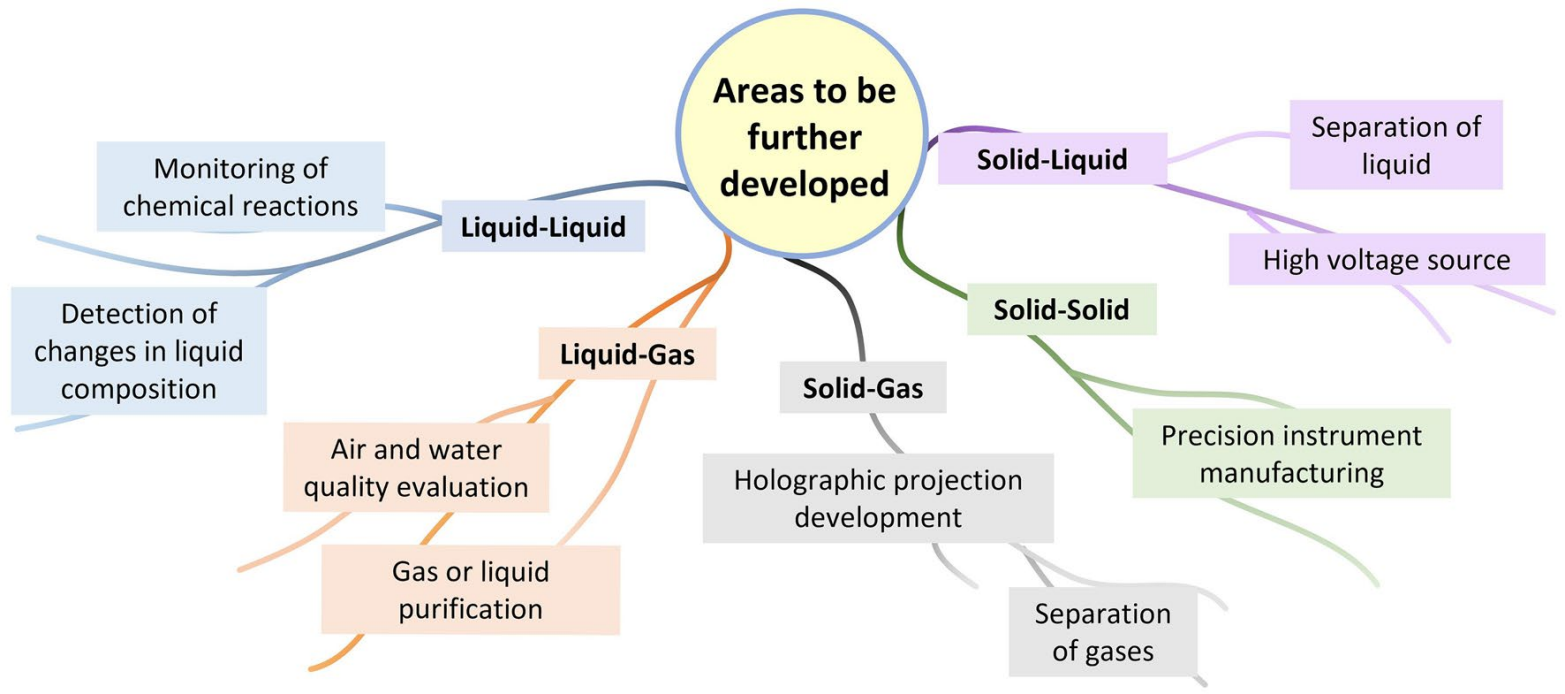
Applications areas to be further developed

Despite the existing technical challenges, contact electrification at different interfaces is already demonstrating great vitality in some emerging applications. In the future, CE at different interfaces will certainly develop their own characteristics and enable a wider variety of applications [158]. Figure 11 illustrates possible future directions. For example, CE at liquid–liquid interfaces offer unlimited possibilities for monitoring chemical reactions and detecting changes in liquid composition, while the challenge of achieving rapid consolidation and separation at liquid–liquid interface remains due to the limitations of existing technology. And the unresolved issue of measuring electrical signals during liquid–liquid contact separations [159]. Recently, the development of inflow-liquid-switching porous nanofiber membranes and Liquid–liquid phase separation in biology have opened up the possibility of rapid consolidation and separation at liquid–liquid interface. Liquid–gas CE can be used to assess and enhance air and water quality. Additionally, solid–gas CE is expected to enable holographic projection techniques and used to separate different gases if effective methods for controlling the erratic diffusion of gases can be found. Research on high-pressure solid–liquid TENG is also in a booming phase. Solid–solid CE, which is the most widely researched, may help in the manufacture of precision instruments and further stimulate the development of CE at other interfaces.

## Conclusions and Prospects

Based on the previous studies of TENG with various interfaces, this work provides an overview of the mechanisms, modification methods, and applications of CE at solid–solid, solid–liquid, solid–gas, liquid–liquid, and liquid–gas interfaces, respectively [29]. The charge generation in the TENG device is primarily the result of the combined effect of electron transfer and ion transfer [87]. And the dominant mechanism is different for different interfaces. The cross-referencing and complementary studies of different interfaces can promote the development of TENG [160]. Multiple factors influence charge transfer, variations in the available materials, processability, and environmental stability across different interfaces are discussed in details, in order to guide a universal approach for interfacial modification [161]. Furthermore, through various modification methods, breakthroughs in enhancing surface charge density and TENG device power can be expected. TENG devices based on CE have excellent design and potential for green energy development and can be tailored to practical applications in different environments due to the characteristics of different interfaces [162].

Despite significant progress in understanding CE between different interfaces at laboratory and pilot scales, there remain several challenges that need to be addressed. Studies targeting CE at solid–gas, liquid–liquid, and liquid–gas interfaces are still in their early stages. There is still no clear answer as to what is the determining factor in charge generation. Due to the intrinsic properties of triboelectric nanogenerators [163], only surface charges can be generated, resulting in low output performance. Solutions for charge escape are still required. In addition, the amorphous [164] nature of liquids and gases hinders modifications for specific interface surfaces and charge density enhancement. Inadequate development of computational systems and circuit management also makes it difficult to implement TENG devices for multifunctional applications. At the same time, the above factors are also obstacles in the way of commercialization of TENG [165].

Through a comprehensive review of previous research, we summarize and propose several potential optimization methods, hoping to facilitate the development of CE.

1. Novel designs and strategies need to be delved to elucidate and enhance interfacial effects, and researches can draw inspiration from other inter-object reaction processes. Further explorations are also required to determine how these effects can be applied to modify interfacial CE.
2. In the quest for novel synthetic materials, researchers also can leverage the potential of natural materials to achieve cost-effectiveness and biological benefits, while minimizing the use of hazardous and non-biodegradable chemicals, and mitigating environmental impact. Development of composite materials with functionality can be explored, harnessing the unique advantages of diverse power generation methods such as piezoelectric, photoelectric, thermoelectric, and magnetoelectric. By combining these methods, materials with additional special properties will be produced.
3. To achieve the desired TENG device, a range of modification methods spanning from the atomic to macroscopic level are required. Sub-microstructural modifications and leverage existing micro–nanoprocessing techniques should be focused on to achieve further microstructural changes. For instance, chemical vapor deposition and magnetron sputtering are techniques capable of forming nanoscale films or coatings on substrates. On the other hand, plasma etching, photolithography, and electron beam exposure techniques excel in creating high-resolution graphics and mapping microfabricated structures. All of these methods have the potential to enhance CE at interfaces. Integration of existing film processing and textile technologies [166], such as co-extrusion, coating, and splitting, can offer novel strategies for processing triboelectric materials.
4. From a macroscopic device structure perspective, power management circuits and charge pumps can enhance the output performance of TENGs, with charge replenishment methods like electric pumps being employed to suppress charge dissipation. Furthermore, the design of TENGs should be application-oriented. Interdisciplinary cooperation should also be emphasized in the promotion of TENG technology [167].

The wide range of materials and structural designs can be used in interface CE for clean energy conversion and more efficient energy storage and release. We hope that our review will offer valuable guidance for future interface designs, enabling a wider range of practical applications and unprecedented performance.
